# Determination of carbohydrate-deficient transferrin in a Han Chinese population

**DOI:** 10.1186/1471-2091-15-5

**Published:** 2014-02-27

**Authors:** Binbin Song, Jing Zhu, Jiong Wu, Chunyan Zhang, Beili Wang, Baishen Pan, Wei Guo

**Affiliations:** 1 Department of Laboratory Medicine, Zhongshan Hospital, Fudan University, Shanghai 200032, China

**Keywords:** CDT, Alcohol biomarker, Capillary electrophoresis, Han Chinese population

## Abstract

**Background:**

Carbohydrate-deficient transferrin (CDT) is a widely used alcohol biomarker. Because of the high prevalence of chronic alcohol abuse in many countries, CDT plays an important role in the areas of traffic, clinical, and forensic medicine. However, CDT levels have not been determined in the Han Chinese population. Therefore, we investigated the frequency of genetic transferrin variants and the relationship between CDT levels and alcohol consumption in this population. From this data, we established a CDT cut-off for Han Chinese and evaluated the analytical performance of the CDT capillary zone electrophoresis system.

**Results:**

The prevalence of transferrin variants was 4.14%. The mean CDT level of the reference group was 0.73%. We recommended CDT level >1.5% as cut off standard of alcohol intake to ensuring the specificity was best. The CDT test total precision for 0.5%, 0.7%, and 1.55% was 14.4%, 11.5%, and 7.2%, respectively. The data showed good linearity in the studied range of 0.6% to 8.2%.

**Conclusions:**

These results demonstrate that CDT is a useful marker to detect heavy daily alcohol consumption. We proposed and evaluated the first CDT cut-off for the Han Chinese population, and we showed that the CDT capillary zone electrophoresis system is a reliable analytic method.

## Background

Alcohol abuse is common throughout the world. Alcoholism causes neurological and psychiatric damage, as well as other somatic disorders. Alcohol is also regarded as one of the most dangerous factors in traffic accidents. The biological, clinical, and social effects of alcohol abuse highlight the urgent need for objective and specific markers for alcohol-related diseases and for early detection of potential alcohol abusers. Laboratory markers of alcohol consumption can play an important role in identifying alcohol intake. However, the traditional biomarkers of alcohol abuse, such as gamma glutamyl transferase (GGT), mean corpuscular volume (MCV), alanine transaminase (ALT) and aspartate transaminase (AST), have variable and limited sensitivity and specificity [1]. Therefore, more attention has been paid to new alcohol markers, especially carbohydrate-deficient transferrin (CDT).

CDT refers to the less sialylated forms of human transferrin: asialo- and disialo-transferrin (which lack one or two complete N-glycans) [2]. It is assumed that alcohol intake ≥50–80 g/day for a period of at least two weeks leads to increased concentrations of CDT [3]. Although the mechanism remains unknown, a large number of studies indicate that CDT is a good biomarker for the diagnosis of heavy alcohol consumption, with higher sensitivity and specificity than any of the other conventional markers [4-7].

Since its discovery in 1976 [8], CDT has quickly become the focus of basic research and clinical studies. Numerous studies have shown that there is a close correlation between CDT levels and alcohol consumption, and the US Food and Drug Administration approved CDT as a biomarker to assess alcohol intake in 2001 [2]. Currently, CDT is recognized as an independent alcohol marker, with reliable sensitivity and specificity to identify heavy drinking [9]. For over 4.5 years, it has been used in traffic medicine among subjects applying for driver’s license renewal or regranting in Belgium [10]. In addition to effectively estimating alcohol consumption, CDT also provides a novel tool to diagnose alcohol-related diseases. Today, CDT has clinical, forensic, and judicial applications. Approximately 200–300 reports on CDT and alcohol have been published, but CDT levels have never been determined in the Han Chinese population. Given the different alcohol customs in China and the West, we aim to establish a CDT cut-off for the Han Chinese population.

## Results

### Normal value of CDT

The nondrinkers and light/moderate drinkers were used to investigate normal CDT values. Table 1 presents CDT values grouped according to gender and three age groups. There was a statistically significant difference between the genders (P = 0.00, <0.05), with male CDT values being higher than those of females in every age group. The differences among the three groups were P = 0.62 for males and P = 0.03 for females. The difference for females between the 0–40-year group and the 41–60-year group were also significant (P = 0.02, <0.05).

**Table 1:** Non-heavy drinkers divided into 6 groups

		**0-40y**		**41-60y**		**61-80y**
Male (n = 299)	Number	115		126		58
	Mean CDT (%)	0.76		0.75		0.78
	CDT SD	0.18		0.18		0.17
	*P*		0.82		0.33	
Female (n = 387)	Number	147		185		55
	Mean CDT (%)	0.73		0.68		0.73
	CDT SD	0.16		0.18		0.17
	*P*		0.02*		0.09	

*The results of statistics analysis between subgroup appear significant.

### Genetic variants of transferrin

Among our original 797 subjects, 33 individuals (4.14%) had genetic variants of transferrin (B or D variants). These variants were detected by capillary electrophoresis, but this method cannot identify the exact type of B or D variant. We also used CDT-IS as a control to exclude false genetic CDT variants caused by monoclonal immunoglobulins in the beta zone. All of the variant results could not be incorporated into the statistical analysis.

### The relationship between CDT levels and alcohol consumption

**Figure 1:**
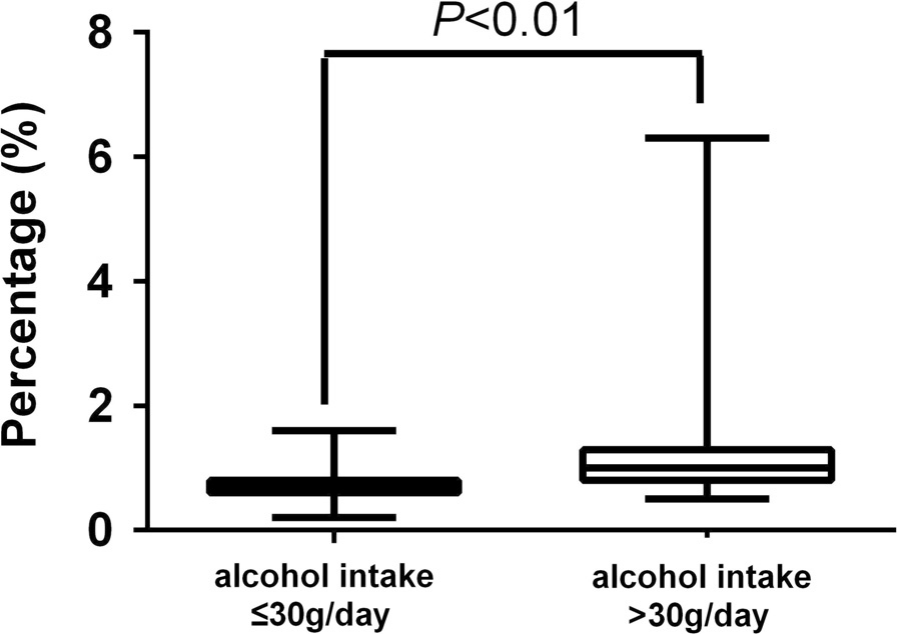
CDT levels and daily alcohol intake data for all subjects.

The relationship between CDT values and daily alcohol intake is shown in Figure 1. Among nondrinkers, CDT values ranged from 0.2% to 1.6%. For drinkers, the lowest CDT value was 0.5% (with a daily alcohol intake of 5 g) and the highest was 6.3% (with a daily alcohol consumption of 240 g).

Based on alcohol consumption, the population was divided into six subgroups. The alcohol consumption and CDT values of all subjects are presented in Table 2. With increasing daily mean alcohol intake, the CDT values raised gradually. This phenomenon was not statistically significant for daily alcohol intake ≤45 g but showed significance when alcohol consumption exceeded this value. A difference of mean %CDT was observed between subgroups 4 and 5 (P = 0.04, <0.05), and between subgroups 5 and 6 (P = 0.000, <0.05). Such a variation in consecutive subgroups only existed between these subgroups.

**Table 2:** Alcohol consumption and %CDT data

Subgroup	1		2		3		4		5		6
(Alcohol g/day)	0		≤15		16-30		31-45		46-60		>60
Number (M-W)	623 (243-380)		31 (26-5)		32 (30-2)		28 (25-3)		28 (26-2)		22 (22-0)
Mean alcohol intake	0.00		9.20		22.80		39.20		55.90		133.40
%CDT mean	0.73		0.75		0.80		0.85		0.90		2.34
%CDT range	0.20-1.60		0.50-1.00		0.60-1.00		0.70-1.10		0.70-1.30		1.00-6.30
*P*		0.59		0.10		0.09		0.04*		0.00*	

*The results of statistics analysis between subgroup appear significant.

### CDT cut-off

**Figure 2:**
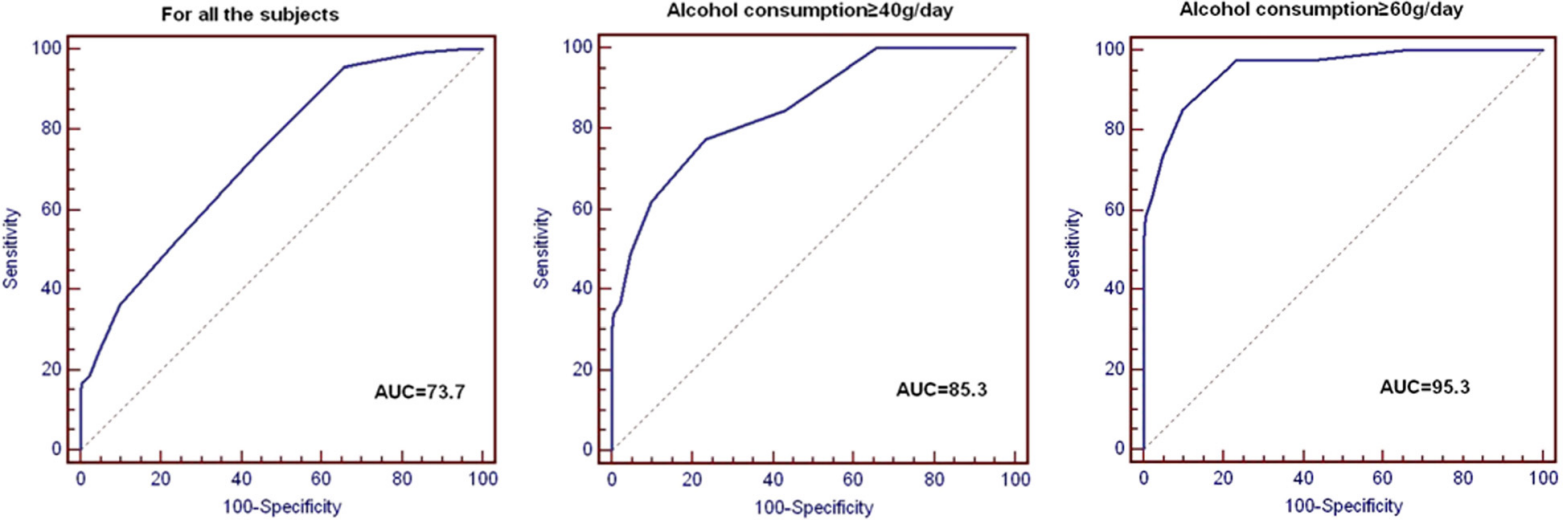
Receiver operating characteristic curves of %CDT according to each target amount of daily alcohol consumption.

We proposed a CDT cut-off of 1.5%, based on the 95^th^ percentile value of drinkers [11]. To evaluate the sensitivity and specificity of the chosen cut-off, receiver operating characteristic (ROC) curves were generated (Figure 2 and Table 3). The area under the ROC curves was largest for the group with alcohol consumption of 60 g/day or more. The cut-off value showed great specificity, but the sensitivity was far from ideal.

**Table 3:** Sensitivities and specificities of 1.5% as cut-off

**Different amount of alcohol consumption (%)**			
	**All the subjects**	**≥ 40 g/day**	**≥ 60 g/day**
Sensitivity (95% CI)	13.5	26.8	46.3
	8.3-20.2	16.9-38.6	30.7-62.6
Sensitivity (95% CI)	99.9	99.9	99.8
	99.1-100	99.1-100	99.1-100

### Precision

The results of the precision studies are shown in Table 4. The intra-run and between-day precision of three serum pools is presented. The maximum discrepancy in the intra-run precision study was 0.2%, 0.1%, and 0.2% for P1, P2, and P3, respectively. Thus, the repeatability of the system for routine testing is acceptable.

**Table 4:** Precision of %CDT using capillary electrophoresis on three different serum pools

**Mean CDT (%)**		**Intra-run precision**	**Between day**	**Total precision**
		**SD (CV%)**	**SD CV (%)**	**CV (%)**
P1	0.50	0.04 (8.00%)	0.06 (12.00%)	14.41
P2	0.70	0.04 (5.70%)	0.07 (10.00%)	11.52
P3	1.55	0.05 (3.20%)	0.10 (6.50%)	7.20

### Linearity

In the linearity experiment, the measured CDT values were similar to the calculated values, with a correlation coefficient of 0.997. The slope of first-order polynomial significantly differed from 0.0 (P = 0.000). The slopes of second-order and third-order polynomials also showed statistically significant differences from 0.0 (P = 0.450 and 0.500, respectively). The CDT detection by the measurement system was linear in the studied range of 0.6% to 8.2%.

## Discussion

Our study used the first large-scale abstinent group to investigate normal CDT values in Han Chinese. The mean CDT value of all non-heavy drinkers was 0.73%. Although the causes remain unknown, our data revealed a small statistically significant difference between genders. The mean CDT value was higher in males than in females. A small statistically significant difference was observed between the various age groups in females but not in males. This finding seems to be consistent with the report by Bergström and Helander [12]. In Jonas P and Anders H study [13], the upper reference limit was set at 1.7%, which is higher than our proposed CDT cut-off of 1.5%. These differences may be due to the measurement systems (high-performance liquid chromatography [HPLC], vs. capillary electrophoresis) and target subjects (Western vs. Asian populations). Many factors influence CDT values, including atypical phenotype distributions of alcohol dehydrogenase [14], amount of daily alcohol consumption, amount of alcohol consumed per drinking session, duration of abstinence before blood sampling, body weight [15] and consumption of fermented food. All these factors may contribute to the CDT differences between Han Chinese and other populations.

Our study is the first to report the frequency of transferrin variants in a large Han Chinese population. Genetic transferrin variants result from amino acids substitutions in the polypeptide chain [16]. Most individuals express allele C, but at least 38 transferrin variants have been found [17]. Although only four isoforms show a prevalence of >1%, transferrin variants can interfere with CDT analysis because of their similar isoelectric points (pIs). transferrin-D can cause false positive results for non-abusers [18], and transferrin-B can cause false negative results for chronic alcohol abusers [2]. These variants can be detected by capillary electrophoresis and HPLC, avoiding with inaccurate CDT calculations. It has been reported that the frequency of genetic transferrin variants is low in Caucasians (<1%) but high in African Americans, Africans, and natives of South America [19,20]. However, there was no precise data for the Han Chinese population. In our study of Han Chinese, 33 of 797 individuals (4.14%) had a transferrin-B or transferrin-D genetic variant.

We also analyzed the correlation between CDT values and alcohol consumption. Some studies found an increase in CDT serum concentrations after sustained alcohol consumption of 50–80 g/day for at least one week, but other reports showed an increase in CDT values after 4–6 times the standard alcohol consumption for at least two weeks [21]. CDT values slowly normalize during periods of abstinence. Although there is a clear correlation between CDT levels and chronic alcohol intake, it remains unclear how CDT values should be used to diagnose alcohol abuse. For instance, it is unclear whether we can extrapolate alcohol consumption (especially moderate alcohol intake of 10–20 g/day) according to CDT values. Table 3 shows that the proportion of individuals in subgroups 2 (<15 g/day), 3 (15–30 g/day), and 4 (31–45 g/day) whose CDT values are below the CDT cut-off is nearly 91%, 85%, and 62%, respectively. Ninety five percent of the group with daily ethanol intake above 60 g/day have CDT levels higher than 1.5%, suggesting that this cut-off can be used to confirm recent drinking behavior. We found a significant threshold in CDT values between subgroups 4 and 5 (*P* < 0.05) (Table 3), suggesting that CDT levels are sensitive to high alcohol intake, such as more than 60 g/day. We found no correlation between CDT values and years of sustained drinking (data not shown). In the Figure 2, receiver operating characteristic curves of CDT according to each target amount of daily alcohol consumption were carried out. The area under ROC curves was best in the alcohol consumption ≥ 60 g/day group. When setting cut-off as 1.5%, the specificities were all above 90%. However, the sensitivities were far from ideal (Table 4). To avoid the numerous biases that can be made during the diagnosis of alcoholism, evaluating CDT with other biomarkers has been recommended.

Furthermore, we evaluated the analytic performance of the Sebia capillary electrophoresis measurement system. The precision and linearity studies showed excellent results. CDT measurements require high precision (especially near the cut-off value) for clinical and forensic applications. Compared with the other studies, our results of total CV were high. This might be because we selected the lower values of CDT serum pools, and our lack of high value CDT serum pools could be a drawback of our study.

## Conclusions

Previous studies have shown that CDT values provide a diagnostic tool to estimate heavy ethanol consumption, especially daily intake over 45 g. Nonetheless, few studies have been conducted to assess this marker in the Chinese population. We evaluated the frequency of genetic transferrin variants, determined the CDT normal range, and proposed a CDT cut-off from a large Chinese population. Further studies focusing on heavy alcoholics in the Chinese population are needed.

## Methods

### Study population

In total, 718 adults (340 males aged 17 to 85 years and 378 females aged 19 to 85 years) were recruited from five Shanghai communities from July to November 2010. Because CDT levels can be influenced by alcohol-independent factors, the following exclusion criteria were used for the abstinent reference population: presence of liver disease (such as hepatitis or cancer), autoimmune liver disease (such as primary biliary cirrhosis), end-stage liver disease, previous liver surgery, liver trauma, and pregnancy. This study was performed with permission from the Ethical Committee of Zhongshan Hospital of Fudan University (Shanghai, China), and written consent was obtained from all participants. The subjects were interviewed about any history of alcoholism and their daily drinking habits (with regard to both the type and amount of alcohol). Simultaneously, 79 surplus samples from subjects with a clinical diagnosis of alcohol disease were obtained from our laboratory to enlarge the non-abstinent group, and these subjects reported their alcohol intake using the same questionnaires. All questionnaires were appropriately preserved. A total of 33 individuals with genetic variants of transferrin were excluded to give a total study population of 764.

According to the World Health Organization/International Society on Biomedical Research on Alcoholism interview schedule, the study population was divided into three groups: “nondrinker” (n = 623), indicated to be totally abstinent or one who drinks alcohol only on special occasions; “light/moderate drinker” (n = 63), one who drinks <30 g ethanol/day for men or <20 g ethanol/day for women; “heavy drinker” (n = 78), one who drinks >30 g ethanol/day for men or >20 g ethanol/day for women. The first two groups were used to establish reference CDT levels. They were further divided into six groups according to gender and age to evaluate the influence of these parameters on CDT levels. The second and third groups were used to show the relationship between alcohol intake and CDT level, and to establish the CDT cut-off at the 95% percentile, following the guidelines in Hock et al. [11]. The demographics of the study population are given in Table 5.

**Table 5:** Demographics of study population (N = 764)

	**Abstinent reference group**	**Nonabstainer group**
	**(N = 623)**	**(N = 141)**
Age (years)	17-85	23-67
Average age (years)	45.17	49.11
Gender (% male)	39.00%	94.33%
Men	243	129
Women	380	12
Smokers	152	72
Hypertension	43	24
Hyperlipidemia	32	12
Heart disease	13	5
Respiratory diseases	12	10
Renal disease	10	0
Thyroid illness	8	0
Alcohol consumption (g/day)		
0	623 (100.00%)	0 (0.00%)
1-15	0 (0.00%)	31 (21.99%)
16-30	0 (0.00%)	32 (22.70%)
31-45	0 (0.00%)	28 (19.86%)
46-60	0 (0.00%)	28 (19.86%)
61-120	0 (0.00%)	12 (8.51%)
>120	0 (0.00%)	10 (7.08%)

In addition, three sample pools (P1, P2, and P3) were used for the precision study. They were analyzed according to the Clinical and Laboratory Standards Institute (CLSI) EP-5 protocol. P1 was prepared to obtain a lower normal CDT level (0.5%), P2 was prepared to obtain a higher normal CDT level (0.7%), and P3 was prepared to obtain a normal CDT level near the cut-off (1.5%). Another two samples, one with a low CDT level (0.6%) and one with a high CDT level (8.2%), were prepared for the CDT linearity measurements.

Venous blood samples were collected under fasting conditions into vacutainer tubes (Becton Dickinson, USA) containing no additives. Serum samples were prepared 5 min after collection by centrifugation at 3500 g and stored at -80°C until testing, a condition under which the transferrin glycoform pattern is known to be stable [22,23].

### Analytical methods

CDT measurements and identification of transferrin genetic variants were performed using the Capillarys 2 capillary zone electrophoresis system (Sebia, France). This instrument performs all operations automatically to obtain a complete transferrin profile for quantitative analysis of CDT. The transferrin isoforms, separated in silica capillaries according to their electrophoretic mobility, were directly detected at an absorbance of 200 nm. Capillary electrophoresis has three advantages: the genetic variants of transferrin are detected, the relative CDT concentrations (percentages of total transferrin) are given, and electrophoresis curves are generated for easier and more accurate data interpretation.

### Precision

The intra-run and between-day precision studies were carried out following CLSI EP5-A protocol using three identical sample pools (P1, P2, and P3). The three samples were analyzed in duplicate 20 times per day to obtain the intra-run precision. Concurrently, these samples were analyzed in duplicate once per day over 20 consecutive days. The mean, standard deviation, and coefficient of variation (CV) were calculated for each sample.

### Linearity

The linearity study was conducted following the CLSI EP6-A protocol. The linearity of CDT measurements was tested between CDT levels of 0.6% and 8.2%. The analysis was performed by mixing variable volumes of two samples containing the same total transferrin concentration (≈1.5 g/L). We calculated the theoretical %CDT values in each dilution based on the original two samples. Each analysis was performed in duplicate.

### Statistical analysis

All statistical analyses were performed using SPSS 16.0 software and Microsoft Excel, with t-tests to assess differences between mean values (P < 0.05 considered statistically significant) and analysis of variance (ANOVA) to assess differences among groups (P < 0.05 considered statistically significant).

## Abbreviations

CDT: Carbohydrate-deficient transferrin; GGT: Gamma glutamyl transferase; MCV: Mean corpuscular volume; ALT: Alanine transaminase; AST: Aspartate transaminase; CLSI: Clinical and laboratory standards institute; CV: coefficient of variation; ANOVA: Analysis of variance; ROC: Receiver operating characteristic; HPLC: High-performance liquid chromatography; pIs: Isoelectric points.

## Competing interests

The authors declare that they have no competing interests.

## Authors’ contributions

BS, JZ and JW carried out the all the subjects questionnaires, sample collection and CDT detection; CZ and BW carried out the initial analysis of the data; BP and WG refined and analyzed the final, submitted structure; BS, BP and WG were primarily responsible for the experimental design, interpretation of the data and writing the manuscript. All authors read and approved of the final manuscript.
